# Jumping Asymmetries and Risk of Injuries in Preprofessional Ballet

**DOI:** 10.1177/03635465231218258

**Published:** 2024-01-22

**Authors:** Niall D.H. MacSweeney, Joseph W. Shaw, George P. Simkin, Charles R. Pedlar, Phil D.B. Price, Ryan Mahaffey, Daniel D. Cohen

**Affiliations:** † Faculty of Sport, Allied Health and Performance Science, St Mary's University, Twickenham, London, UK; ‡ Healthcare Team, The Royal Ballet School, London, UK; § Ballet Healthcare, The Royal Ballet, London, UK; ‖ Global Data and Analytics, Product Safety and Compliance, Amazon, London, UK; ¶ Institute of Sport, Exercise and Health, University College London, London, UK; # Department of Physical Education and Sport Sciences, University of Limerick, Limerick, Ireland; Investigation performed at The Royal Ballet School, London, UK

**Keywords:** injuries, jumping, limb asymmetry, dance, risk factors, biomechanics, knee injury, ankle injury, foot injury

## Abstract

**Background:**

Preprofessional ballet dancers are exposed to the risk of injuries, primarily in the lower extremities, with most injuries occurring during jumping and landing activities. Interlimb asymmetry during jumping and landing activities has been associated with the injury risk in adolescent athletes, but this has not been examined in dancers.

**Purpose:**

To investigate associations between interlimb asymmetry during a double-leg countermovement jump (DL-CMJ) and single-leg jump (SLJ) and the injury risk in adolescent preprofessional ballet dancers.

**Study Design:**

Cohort study; Level of evidence, 2.

**Methods:**

Adolescent preprofessional ballet dancers (N = 255) performed 3 DL-CMJs and 3 SLJs on force plates during annual screening. Absolute and directional (separate values for left and right limb dominance) asymmetries in a set of kinetic variables during a DL-CMJ and in jump height during an SLJ were calculated. Each variable was characterized as having “high” or “normal” asymmetry according to the percentage of asymmetry (greater than or less than or equal to, respectively, the mean ± 0.5 SD) based on the present sample. Risk ratios (RRs) and 95% CIs were calculated based on the injury incidence in the subsequent academic year.

**Results:**

Of the 242 dancers that satisfied the inclusion criteria, 128 injuries were observed in the subsequent academic year. In the whole sample, 3 absolute, 7 left limb–dominant, and 1 right limb–dominant kinetic asymmetry in the eccentric, concentric, and landing phases of the DL-CMJ as well as left limb–dominant jump height asymmetry in the SLJ were associated with a significant (*P* < .001) increase in the injury risk (RR, 1.28-1.69 [95% CI, 1.02-2.37]). Separating by sex, asymmetries in the eccentric and landing phase of the DL-CMJ were not significant in boys, while in girls, RRs for asymmetries in the eccentric and landing phase of the DL-CMJ increased, and SLJ jump height asymmetry was not significant.

**Conclusion:**

Higher asymmetries in certain kinetic variables during the DL-CMJ and in jump height during the SLJ were associated with an elevated risk of injuries in elite preprofessional ballet dancers with some sex-specific differences. Associations were mainly identified for high left limb–dominant asymmetry in the takeoff phase, suggesting that the injury risk may be specific to a relative right limb deficit.

Professional ballet is extremely physical and technically demanding.^41^ Technical ballet training and performances involve slow, controlled movements at a lower intensity with bursts of intermittent, higher intensity activities such as jumping.^25^ Dancers train at vocational schools as preprofessionals from as young as 9 years old, training between 20 and 30 hours per week.^5,6,10,45^ These high training volumes expose preprofessional dancers to the risk of injury,^12^ with the majority of injuries in the lower extremities occurring during jumping and landing activities.^1,31^ Injuries influence dancers’ ability to train, and therefore achieve their professional ambitions, and may have other longer term musculoskeletal consequences.^38^ Reducing the injury incidence is therefore a primary goal for practitioners working with preprofessional and professional ballet dancers.

During ballet performances, professional dancers can complete up to 14 jumps per minute, involving high levels of technical mastery.^42^ Preprofessional dancers undergo a large volume of jump training to be able to reach the standards of the senior level.^19^ Balletic jumps demand large levels of force production for takeoff and for attenuating ground-reaction forces when landing. The technical and aesthetic demands of ballet may lead dancers to favor specific limbs to maximize quality. Consistent preference of 1 limb during training and performances may expose dancers to increased stress on the dominant limb or lead to relative weakness on the contralateral limb. Limb imbalance has been quantified as a percentage of asymmetry, a factor that has been associated with the injury risk in studies on other sports.^11,36^ However, associations between jump-landing asymmetries and the injury risk have not been reported in dancers. Moreover, there is a paucity of research available that associates any physical qualities with the injury risk for preprofessional ballet dancers.^29^

In high-performance settings, the double-leg countermovement jump (DL-CMJ), executed on dual force platforms, is a commonly used to assess strength qualities or “neuromuscular performance” and to simultaneously evaluate interlimb asymmetries in the eccentric (downward), concentric (upward), and landing phases.^8^ However, in settings without force platforms, the single-leg jump (SLJ) is a more accessible method to quantify interlimb asymmetry because of the range of cheaper equipment that can reliably measure jump height.^21,44^ It is unclear whether kinetic asymmetries during the DL-CMJ and SLJ jump height asymmetries have similar associations with the injury risk, as these asymmetries often do not align.^7,39^ To our knowledge, both approaches have not been concurrently examined in the same study.

The purpose of this study was therefore to investigate associations between the injury risk and interlimb asymmetries in a comprehensive set of kinetic variables during a DL-CMJ and in SLJ jump height in adolescent preprofessional ballet dancers.

## Methods

### Participants

A total of 255 participants took part in jump testing as part of their annual screening, and written informed consent was obtained for the use of data in the present analysis from participants and parents. Ethical approval was obtained from the ethics board at St Mary's University, Twickenham, in accordance with the Declaration of Helsinki. All participants were preprofessional, and all trained at the same ballet school (The Royal Ballet School, London). Participants were informed that data would be used for research and disseminated to improve dancers’ health. The dancers’ training schedule corresponded with a normal British academic school year, and specific training demands were categorized by the participant's sex and year group (Table 1). Participants were excluded from the study if they had a current lower limb injury at the time of jump testing or if they left the school during the academic year after initial screening. All data were removed for excluded participants.

**Table 1:** Participants’ Characteristics

Year Group (age, y)	n	Training,^*a*^ h/wk
Year 7 (11-12)		
Male	19	18.8
Female	26	18.8
Year 8 (12-13)		
Male	12	20.7
Female	14	20.0
Year 9 (13-14)		
Male	10	21.1
Female	18	21.1
Year 10 (14-15)		
Male	14	21.5
Female	16	21.5
Year 11 (15-16)		
Male	12	21.3
Female	13	21.3
Year 12 (16-17)		
Male	19	23.0
Female	21	23.0
Year 13 (17-18)		
Male	17	26.0
Female	13	26.0
Year 14 (18-19)		
Male	11	29.3
Female	7	29.3
Total		
Male	114	22.7 (mean)
Female	128	22.7 (mean)

a Approximate training hours were calculated by using the annual weekly timetable template. Hours were likely to vary depending on a performance/rehearsal.

### Data Collection

The present analysis involved jump assessments administered during the first week of 2 consecutive academic years (September 2018 and September 2019) and injury data collected during the whole school year until the final days of the academic term (July 2019 and July 2020, respectively). The majority of data from participants (195/242) that formed the analysis were from the 2018-2019 school year, as the only data included from the 2019-2020 school year were from dancers who were new to the school and were not part of the previous year. Overall, 6 chartered physical therapists collected injury data, which consisted of participant information, injury diagnosis, injury location, injury mechanism, days restricted from full dance practices, and days fully off all dance practices.

Injuries occurring in the corresponding school year after jump testing that affected the lower back and pelvis and any structures inferior to these were included in the analysis. An injured dancer was only included once in the analysis, regardless of the number of additional injuries. A “moderate” threshold, defined as “any anatomic tissue level impairment that resulted in full time loss or a restriction from activity for seven or more days,”^9^ was used to define an injury. Dancers who only had injuries below this threshold were therefore categorized as “noninjured.” The number of days of restricted activity or time lost from activity was determined from the first date on which the dancer reported the injury to the physical therapist until the therapist removed all restrictions from full participation in classes. Therefore, a dancer who sustained a minor injury (<7 days of activity restriction) was included in the study but classified as noninjured.

**Figure 1.**
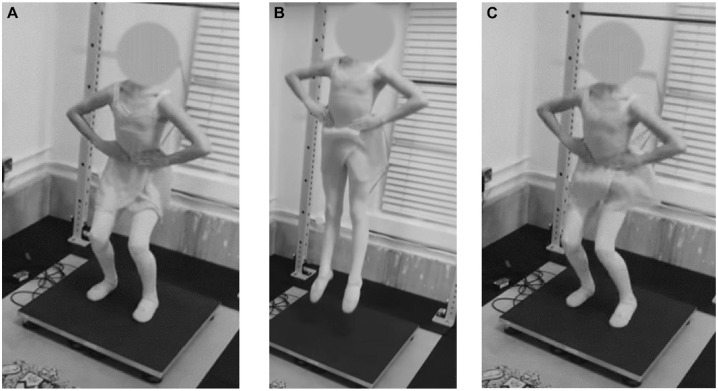
Countermovement jump. (A) Participants moved from a standing position into a bent knee position as part of the countermovement section of the jump. (B) Participants jumped as high as possible. (C) Participants then landed with each foot on each force plate. Hands were maintained on the hips throughout.

All participants performed 3 bilateral countermovement jumps (DL-CMJs) with a 5-second pause between each repetition. Jumps were performed on FD4000 (VALD Performance) and PASPORT (PASCO scientific) force plates with 1 leg on 1 force plate. Data were acquired via ForceDecks software (VALD Performance) with a sample rate of 1000 Hz. Before measurements, a standardized warm-up was performed, consisting of 3 DL-CMJs, followed by 3 SLJs. Participants were instructed to jump as high as possible with their hands on their hips and to land on the force plates (Figure 1). The process was then repeated for the left and right legs for the SLJs, with 3 jumps performed on the left leg, followed by 3 on the right leg.

### Data Analysis

Raw force-time data were exported and kinetic asymmetries analyzed using Python (Version 3.10.01; Python Software Foundation). Descriptions of the kinetic variables can be found in Appendix 1 (available in the online version of this article). Asymmetries for all kinetic variables were calculated using the bilateral strength asymmetry formula^20^:

$$\frac{\left(\mathrm{Stronger}\;\mathrm{Limb}-\mathrm{Weaker}\;\mathrm{Limb}\right)}{\mathrm{Stronger}\;\mathrm{Limb}}\;\times 100.$$

For all variables, the higher value of the 2 limbs was used as the stronger limb. Absolute asymmetries ignored the direction of asymmetry. Directional asymmetries were derived from the same calculation as absolute asymmetries, but the direction (ie, dominance) was also expressed. The analysis was conducted on a variable-by-variable basis, such that “dominance” was defined for each variable and not for the individual participant. An example of this calculation can be seen in Appendix 2 (available online).

Asymmetries were defined as “high” or “normal” according to whether the value was greater than or less than or equal to, respectively, the variable's mean ± 0.5 SD:

$$\begin{array}{c} >\mathrm{Mean}\;\mathrm{Asymmetry}\;\%\;+\left(0.5*\mathrm{SD})\right)\;\mathrm{and}\\ \leq \mathrm{Mean}\;\mathrm{Asymmetry}\;\%\;+\left(0.5*\mathrm{SD})\right),\;\mathrm{respectively}. \end{array}$$

### Statistical Analysis

Because of the lack of previous research in this area, an exploratory analysis^27^ was performed on all kinetic variables for absolute and directional asymmetries for male and female participants. To do this, participants’ left limb–dominant, right limb–dominant, and absolute asymmetry percentage value for each variable during the DL-CMJ and for jump height during the SLJ was individually dichotomized as high or normal. After this, risk ratios (RRs) were calculated to describe the probability of injuries for those with high relative to normal asymmetries. A significant association between asymmetry and the injury risk was indicated by 95% CIs that did not cross 1.

## Results

### Participants’ Characteristics

The final analysis included 242 participants, of whom 128 suffered ≥1 injury during the study period. All data collected from the 13 participants who withdrew from the study were removed from the analysis. Participants’ characteristics can be found in Table 1. Descriptive injury data are displayed in Table 2.

**Table 2:** Injury Data

	Value
No. of injuries, n	128
Left-sided	54
Right-sided	58
Bilateral	13
Central^*a*^	3
Tenogenic	22
Arthrogenic	51
Myogenic	29
Osteogenic	26
Mean time from jump testing to injury, d, mean ± SD	125.6 ± 88.6
Median time from jump testing to injury, d, n	121

a A central injury is one that occurred on either side of the spine/sternum.

### Asymmetry and Injury Risk

**Figure 2.**
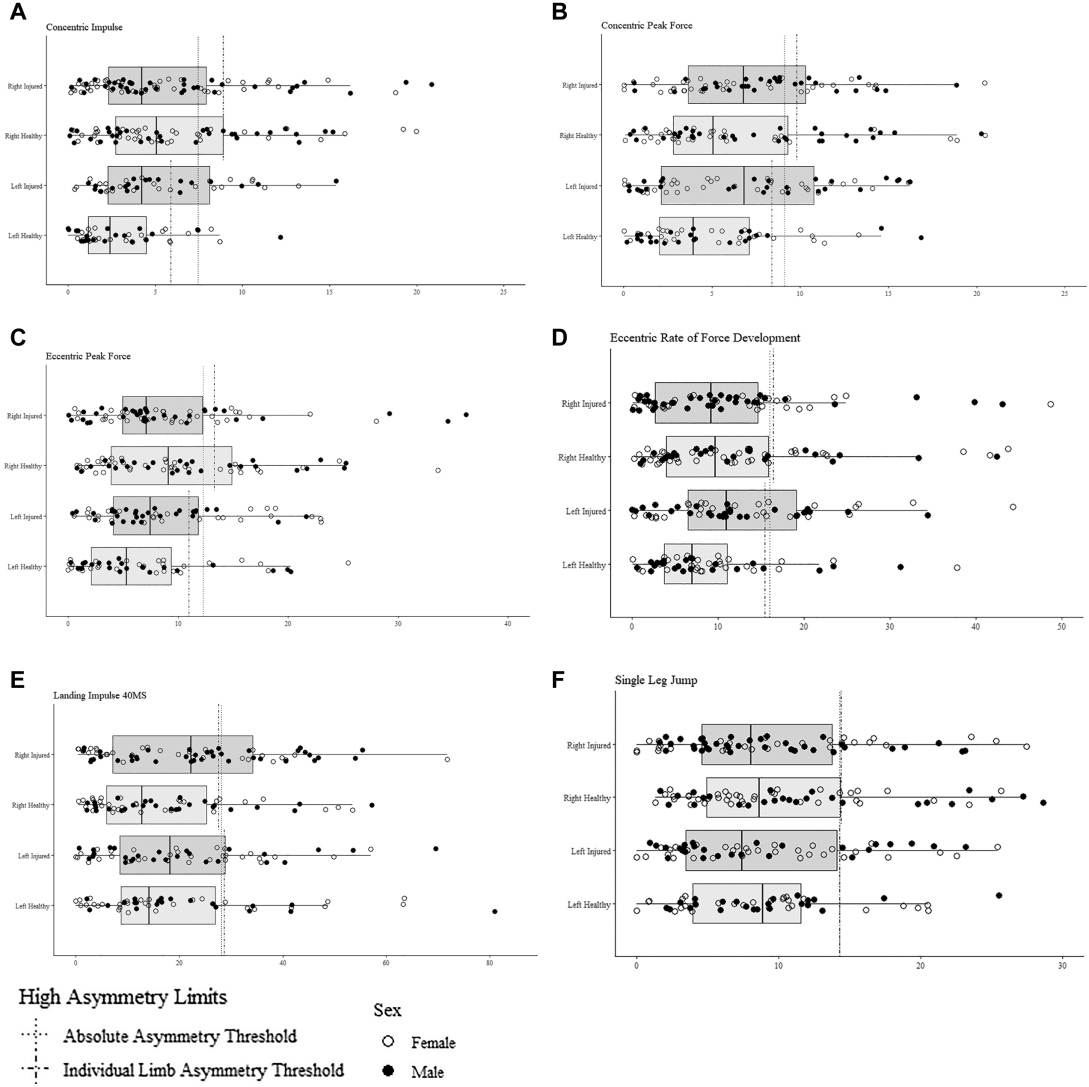
Distribution of participants for significant kinetic variables.

During the DL-CMJ, the majority of participants displayed right limb dominance in eccentric and concentric phase variables and left limb dominance in landing phase variables as well as right limb dominance in jump height during the SLJ. Contingency tables displaying the number of dancers with high and low asymmetry for each variable and each limb can be seen in Appendix 3 (available online). Variables for which high asymmetry was significantly associated with an increased injury risk (all *P* < .001) were left limb–dominant eccentric peak force (RR, 1.45 [95% CI, 1.02-2.05]), eccentric rate of force development (RFD) (RR, 1.60 [95% CI, 1.15-2.23]), concentric impulse (RR, 1.58 [95% CI, 1.16-2.15]), concentric peak force (RR, 1.60 [95% CI, 1.18-2.16]), concentric impulse 100 ms (RR, 1.69 [95% CI, 1.20-2.37]), concentric impulse part 1 (RR, 1.65 [95% CI, 1.19-2.28]), and concentric impulse part 2 (RR, 1.71 (1.17-2.48). Absolute asymmetries were significantly associated with an increased injury risk in concentric peak force (RR, 1.28 [95% CI, 1.01-1.62]), landing RFD 40 ms (RR, 1.29 [95% CI, 1.02-1.64]), and landing impulse 40 ms (RR, 1.31 [95% CI, 1.03-1.67]). Right limb–dominant asymmetry in landing impulse 40 ms (RR, 1.40 [95% CI, 1.02-1.91]) also demonstrated significant associations with the injury risk. In addition, having left limb–dominant (right limb deficit) asymmetry in jump height during the SLJ was significantly associated with the injury risk (RR, 1.48 [95% CI, 1.08-2.03]). Of the variables measured during the DL-CMJ, 3 absolute, 7 left limb–dominant, and 1 right limb–dominant asymmetries were associated with a significant increase in the injury risk. The distribution of individual participants’ values for significant kinetic variables is shown in Figure 2, and a full list of the distribution can be seen in Appendix 3. The RRs and 95% CIs associated with high asymmetries and lower limb injuries can be found in Table 3.

**Table 3:** Kinetic Variable Asymmetries for All Dancers^*a*^

	Absolute	Left	Right
Double-leg countermovement jump			
Eccentric (downward) phase			
Minimum force	0.98 (0.72-1.34)	1.23 (0.82-1.87) [n = 129]	0.88 (0.59-1.32) [n = 113]
Yielding RFD	0.87 (0.65-1.16)	0.78 (0.51-1.20) [n = 112]	0.85 (0.56-1.29) [n = 130]
Deceleration RFD	1.00 (0.14-0.77)	1.10 (0.79-1.55) [n = 113]	0.89 (0.58-1.36) [n = 129]
RFD	1.12 (0.85-1.47)	**1.60 (1.15-2.23) [n = 107]**	0.84 (0.55-1.30) [n = 135]
Deceleration impulse	0.85 (0.62-1.15)	0.93 (0.61-1.42) [n = 108]	0.81 (0.52-1.26) [n = 134]
Peak force	1.01 (0.77-1.33)	**1.45 (1.02-2.05) [n = 107]**	0.78 (0.52-1.18) [n = 135]
Force at 0 velocity	1.01 (0.77-1.32)	1.36 (0.96-1.93) [n = 108]	0.76 (0.50-1.15) [n = 134]
Concentric (upward) phase			
Impulse 100 ms	1.21 (0.94-1.55)	**1.69 (1.20-2.37) [n = 105]**	0.89 (0.60-1.30) [n = 137]
Impulse part 1	1.17 (0.90-1.51)	**1.65 (1.19-2.28) [n = 108]**	0.91 (0.61-1.35) [n = 134]
Impulse part 2	1.09 (0.84-1.42)	**1.71 (1.17-2.48) [n = 90]**	0.91 (0.65-1.31) [n = 152]
Peak force	**1.28 (1.01-1.62)**	**1.60 (1.18-2.16) [n = 107]**	1.15 (0.80-1.64) [n = 135]
Force at peak power	1.07 (0.82-1.40)	1.42 (0.98-2.07) [n = 100]	0.80 (0.55-1.18) [n = 142]
Impulse	1.14 (0.88-1.48)	**1.58 (1.16-2.15) [n = 98]**	1.00 (0.68-1.48) [n = 144]
Landing phase			
Impulse 40 ms	**1.31 (1.03-1.67)**	1.12 (0.77-1.64) [n = 113]	**1.40 (1.02-1.91) [n = 129]**
RFD 40 ms	**1.29 (1.02-1.64)**	1.21 (0.84-1.73) [n = 116]	1.35 (0.98-1.85) [n = 126]
Mean RFD	1.12 (0.87-1.46)	1.23 (0.90-1.70) [n = 115]	1.08 (0.73-1.59) [n = 127]
Peak force	1.88 (0.83-1.42)	1.25 (0.88-1.79) [n = 121]	0.95 (0.63-1.42) [n = 121]
Impulse	1.09 (0.84-1.42)	0.93 (0.60-1.45) [n = 112]	1.27 (0.93-1.73) [n = 130]
Single-leg countermovement jump			
Jump height	1.20 (0.94-1.54)	**1.48 (1.08-2.03) [n = 111]**	1.01 (0.69-1.46) [n = 131]

a Data are shown as risk ratio (95% CI). RFD, rate of force development. n, number of dancers characterised with the relevant limb dominance. **Data in bold** indicates significant injury association.

When participants were split by sex, there was a difference in the variables that displayed significant relationships with the injury risk. For male dancers (Table 4), asymmetries in left limb–dominant eccentric minimum force (RR, 1.57 [95% CI, 1.06-2.32]), concentric impulse 100 ms (RR, 1.90 [95% CI, 1.19-3.04]), concentric impulse part 1 (RR, 1.57 [95% CI, 1.03-2.39]), concentric peak force (RR, 1.71 [95% CI, 1.16-2.53]), concentric impulse (RR, 1.90 [95% CI, 1.19-3.04]), and jump height (RR, 1.81 [95% CI, 1.18-2.76]) showed significant associations with the injury risk. No absolute or right limb–dominant asymmetries shared this association for male dancers. For female dancers (Table 5), asymmetries in left limb–dominant eccentric peak force (RR, 1.72 [95% CI, 1.05-2.84]), eccentric RFD (RR, 1.72 [95% CI, 1.03-2.82]), concentric impulse 100 ms (RR, 1.69 [95% CI, 1.04-2.84]), concentric impulse part 1 (RR, 1.80 [95% CI, 1.09-2.97]), and concentric impulse part 2 (RR, 1.78 [95% CI, 1.11-2.86]) were all significantly associated with the injury risk alongside absolute asymmetry in landing RFD 40 ms (RR, 1.47 [95% CI, 1.05-2.08]), concentric impulse 100 ms (RR, 1.44 [95% CI, 1.02-2.03]), and concentric impulse part 1 (RR, 1.44 [95% CI, 1.02-2.03]).

**Table 4:** Kinetic Variable Asymmetries for Male Dancers^*a*^

	Absolute	Left	Right
Double-leg countermovement jump			
Eccentric (downward) phase			
Minimum force	1.21 (0.87-1.68)	**1.57 (1.06-2.32) [n = 59]**	1.04 (0.64-1.69) [n = 55]
Yielding RFD	0.92 (0.62-1.36)	0.86 (0.52-1.43) [n = 50]	0.87 (0.46-1.66) [n = 64]
Deceleration RFD	0.97 (0.68-1.38)	0.96 (0.60-1.54) [n = 54]	0.96 (0.56-1.65) [n = 60]
RFD	1.09 (0.75-1.58)	1.50 (0.96-2.35) [n = 52]	0.75 (0.38-1.48) [n = 62]
Deceleration impulse	0.93 (0.62-1.38)	0.71 (0.36-1.40) [n = 53]	0.83 (0.45-1.50) [n = 61]
Peak force	0.85 (0.55-1.29)	1.24 (0.76-2.03) [n = 51]	0.71 (0.39-1.29) [n = 63]
Force at 0 velocity	0.88 (0.59-1.32)	1.21 (0.74-1.97) [n = 50]	0.72 (0.40-1.32) [n = 64]
Concentric (upward) phase			
Impulse 100 ms	1.05 (0.73-1.51)	**1.90 (1.19-3.04) [n = 49]**	0.95 (0.58-1.54) [n = 65]
Impulse part 1	0.93 (0.62-1.38)	**1.57 (1.03-2.39) [n = 52]**	0.78 (0.45-1.37) [n = 62]
Impulse part 2	1.20 (0.85-1.68)	1.59 (0.85-2.96) [n = 40]	1.06 (0.70-1.58) [n = 74]
Peak force	1.17 (0.85-1.62)	**1.71 (1.16-2.53) [n = 50]**	0.96 (0.57-1.60) [n = 64]
Force at peak power	1.02 (0.70-1.48)	1.45 (0.80-2.64) [n = 43]	0.88 (0.55-1.42) [n = 71]
Impulse	1.05 (0.73-1.51)	**1.90 (1.19-3.04) [n = 44]**	0.95 (0.58-1.54) [n = 70]
Landing phase			
Impulse 40 ms	1.28 (0.93-1.75)	1.05 (0.60-1.84) [n = 46]	1.44 (0.98-2.13) [n = 68]
RFD 40 ms	1.13 (0.82-1.55)	0.97 (0.56-1.67) [n = 46]	1.20 (0.80-1.79) [n = 68]
Mean RFD	1.07 (0.76-1.52)	1.09 (0.70-1.69) [n = 48]	1.08 (0.64-1.80) [n = 66]
Peak force	1.04 (0.73-1.49)	1.12 (0.67-1.87) [n = 55]	0.97 (0.59-1.60) [n = 59]
Impulse	1.03 (0.72-1.46)	1.00 (0.50-2.01) [n = 48]	1.08 (0.73-1.58) [n = 68]
Single-leg countermovement jump			
Jump height	1.12 (0.80-1.58)	**1.81 (1.18-2.76) [n = 51]**	0.75 (0.43-1.29) [n = 63]

a Data are shown as risk ratio (95% CI). RFD, rate of force development. n = number of dancers characterised with the relevant limb dominance. **Data in bold** indicates significant injury association.

**Table 5:** Kinetic Variable Asymmetries for Female Dancers^*a*^

	Absolute	Left	Right
Double-leg countermovement jump			
Eccentric (downward) phase			
Minimum force	0.56 (0.26-1.20)	0.60 (0.18-1.99) [n = 70]	0.63 (0.28-1.44) [n = 58]
Yielding RFD	0.84 (0.55-1.27)	0.69 (0.34-1.40) [n = 62]	0.83 (0.50-1.44) [n = 66]
Deceleration RFD	1.02 (0.68-1.53)	1.26 (0.78-2.04) [n = 59]	0.83 (0.43-1.58) [n = 69]
RFD	1.16 (0.79-1.71)	**1.72 (1.05-2.82) [n = 55]**	0.94 (0.53-1.66) [n = 73]
Deceleration impulse	0.77 (0.48-1.24)	1.26 (0.70-2.25) [n = 55]	0.79 (0.41-1.51) [n = 73]
Peak force	1.19 (0.83-1.73)	**1.72 (1.04-2.84) [n = 56]**	0.86 (0.48-1.53) [n = 72]
Force at 0 velocity	1.15 (0.79-1.66)	1.56 (0.94-2.59) [n = 58]	0.80 (0.44-1.43) [n = 70]
Concentric (upward) phase			
Impulse 100 ms	**1.44 (1.02-2.03)**	**1.69 (1.03-2.77) [n = 56]**	1.04 (0.61-1.78) [n = 72]
Impulse part 1	**1.44 (1.02-2.03)**	**1.80 (1.09-2.97) [n = 56]**	1.04 (0.59-1.82) [n = 72]
Impulse part 2	1.02 (0.69-1.50)	**1.78 (1.11-2.86) [n = 50]**	0.80 (0.44-1.45) [n = 78]
Peak force	1.38 (0.97-1.96)	1.48 (0.93-2.37) [n = 57]	1.37 (0.83-2.25) [n = 71]
Force at peak power	1.15 (0.79-1.66)	1.40 (0.87-2.26) [n = 57]	0.76 (0.41-1.40) [n = 71]
Impulse	1.24 (0.86-1.79)	1.36 (0.89-2.08) [n = 54]	1.06 (0.56-1.99) [n = 74]
Landing phase			
Impulse 40 ms	1.34 (0.94-1.91)	1.19 (0.71-1.98) [n = 65]	1.31 (0.78-2.19) [n = 63]
RFD 40 ms	**1.47 (1.05-2.08)**	1.43 (0.90-2.28) [n = 68]	1.52 (0.91-2.54) [n = 60]
Mean RFD	1.16 (0.79-1.71)	1.38 (0.87-2.19) [n = 61]	1.07 (0.59-1.94) [n = 67]
Peak force	1.12 (0.74-1.69)	1.38 (0.85-2.25) [n = 66]	0.89 (0.46-1.75) [n = 62]
Impulse	1.16 (0.79-1.69)	0.88 (0.50-1.57) [n = 64]	1.50 (0.90-2.49) [n = 64]
Single-leg countermovement jump			
Jump height	1.29 (0.90-1.84)	1.23 (0.76-1.99) [n = 60]	1.39 (0.82-2.36) [n = 68]

a Data are shown as risk ratio (95% CI). RFD, rate of force development. n, number of dancers characterised with the relevant limb dominance. **Data in bold** indicates significant injury association.

## Discussion

This study determined associations between interlimb asymmetry in a comprehensive set of kinetic variables during a DL-CMJ and in SLJ jump height with the injury risk in preprofessional ballet dancers aged 11 to 19 years. In the whole sample, high asymmetry in specific kinetic variables during the 3 phases of the DL-CMJ and SLJ jump height during annual screening was associated with up to a 69% higher risk of injuries during the subsequent 9-month school year. The comprehensive analysis of kinetic asymmetries during the DL-CMJ in relation to the injury risk^3,9^ allowed the identification of specific asymmetry-risk associations not previously detected. Notably, 7 significant associations were found using directional asymmetry analysis, with 7 of these involving only high left limb–dominant asymmetry (ie, a greater right limb deficit): eccentric peak force, eccentric RFD, concentric impulse, concentric peak force, concentric impulse 100 ms, concentric impulse part 1, concentric impulse part 2, and jump height. In contrast, landing impulse 40 ms with right limb dominance was significantly associated with the injury risk. Importantly, despite the larger sample size in the absolute asymmetry analysis, only 3 variables were significantly associated with the injury risk: concentric peak force, landing impulse 40 ms and landing RFD 40 ms. The significant associations observed were principally driven by the elevated probability of injuries in participants with high asymmetry; for example, in specific variables, more than 2 of 3 of those with high asymmetry were injured. In contrast, just under 1 of 2 of those classified as having normal asymmetry were also injured. This is reflected by the substantially higher specificity than sensitivity of the significant variables (0.78-0.91 and 0.31-0.50, respectively). As such, this aligns with the complex and multifactorial nature of the injury risk and demonstrates that having low asymmetry in specific variables does not determine the injury risk. However, the high specificity demonstrates that jump-landing asymmetries represent a potentially modifiable risk factor to screen for and address as part of risk reduction strategies, at least in the present population.

### Limb Asymmetry and Ballet

To our knowledge, this is the first study to examine associations between asymmetries in kinetic variables during the DL-CMJ or in jump height during the SLJ and the injury risk in dance. The elevated risk associated with higher left limb–dominant asymmetry during the takeoff phase and right limb–dominant asymmetry during landing could be related to the nature of balletic activity. Kimmerle^23^ highlighted a preference for dancers to use their right leg in powerful activities such as turning and jumping, aligning with other evidence suggesting a right limb bias in ballet training.^2,16,35^ Traditionally, young dancers begin to learn at the barre with their left leg as the “supporting” leg and their right leg as the “gesture” leg.^2^ This may lead to interlimb differences in motor proficiency. However, 2 studies investigated the *grand jeté* jump in preprofessional dancers and found no significant differences in jump height regardless of the takeoff leg used.^14,43^ Despite this, Wyon et al^43^ did identify greater knee flexion in the right limb during the takeoff and landing phases, and Golomer et al^15^ observed a significant relationship between muscle mass in the right limb and jump height, which was not observed contralaterally. These findings correspond with the present population in which dancers were right limb dominant in the majority of variables.

The injury risk may be heightened by the design of practices and performances that directly or indirectly favor the best aesthetic to be produced by the majority of dancers rather than the minority. For instance, Baker and Wilmerding^2^ observed that the majority of activities during ballet classes for both beginner- and advanced-level dancers were taught to favor the use, or more frequent use, of the right side. This type of protocol forces left limb–dominant dancers to use their weaker (right) leg as the lead leg, thereby increasing the relative demands of these activities on the limb, which in turn may drive the greater injury risk observed in dancers with large magnitudes of left limb dominance (relative right-sided deficit). Similarly, in a prospective study of youth athletes in mixed team sports (N = 81), Fort-Vanmeerhaeghe et al^11^ found significantly higher (*P* < .001) SLJ jump height asymmetry in injured than noninjured athletes and suggested that the less dominant limb might have lower “tolerance capacity,” increasing the likelihood of exceeding that tolerance and becoming injured. In the present study, however, the injury incidence in the dancers was similar between limbs (Table 2), challenging a simple explanation with respect to dominance and the risk of injuries.

Managing the symmetry of dance practices and performances by implementing more left limb–dominant training might help to mitigate some of the asymmetries that are present in preprofessional ballet dancers^35^ and better suit those who are more dominant in their left limb. Shaw et al^37^ validated the use of an accelerometery algorithm to monitor ballet-specific jump height and frequency. This type of approach could be used to examine the demands placed on each individual limb to provide insight into relative balance in training and potentially modify accordingly. Where this is not possible, targeted conditioning of the less dominant limb might provide an alternative solution.

### Direction-Specific Findings

One of the strengths of this analysis was the size of the present sample, which enabled the use of internal descriptive data routinely collected by the school as representative of the population and allowed further exploration of directional asymmetry. The finding that the injury risk was associated with a relative deficit in the dominant limb aligns with a study on elite youth soccer players, which observed a significant association between lower right limb–dominant, but not left limb–dominant, vertical ground-reaction forces during an SLJ and the injury risk^33^ (the majority of players were right-footed). Absolute peak force asymmetries during the SLJ also displayed significant relationships (*P* < .001) with the injury risk.

Asymmetry-risk studies have generally considered absolute asymmetry but not the direction of asymmetry.^11,36^ An exception is Malaver-Moreno et al,^30^ who examined military cadets and the risk of medial tibial stress syndrome as an outcome; the current study's findings broadly align with theirs in that left limb–dominant (deceleration RFD in the eccentric phase) asymmetry identified during a DL-CMJ as part of a preparticipation assessment was associated with the injury risk, while right limb–dominant asymmetry was not. Similarly, this study found a significant association between left limb–dominant asymmetry in eccentric RFD and the injury risk, but this association was not evident for absolute or right limb–dominant asymmetry.

These direction-specific findings may have implications for the analysis and interpretation of asymmetry-risk data in other cohorts, providing greater evidence of the injury risk not identified by only examining associations with absolute asymmetry, which is the most commonly used approach for asymmetry analysis.

### Sex Differences

Some potentially important sex differences in the asymmetry-risk associations emerged from the analysis of boys and girls separately. Examining the boys alone, RRs for associations between DL-CMJ concentric, SLJ jump height asymmetries and the injury risk were similar to or greater then observed in the whole sample. In contrast, eccentric RFD and peak force, concentric impulse part 2 and landing asymmetry-risk associations significant in the whole sample and in girls, became non significant in boys. Conversely, in girls SLJ jump height asymmetry was non significant.

Previous evidence suggests that sex differences in jumping asymmetries may influence the injury risk. For instance, concentric peak force asymmetry was associated with the injury risk in boys but not girls, aligning with a recent study by Koźlenia et al.^26^ They found that the injury risk was associated with asymmetry in “peak force” during a DL-CMJ in a sample of active, young adult male participants but not female participants; peak force typically occurs in the concentric (upward) phase and therefore is equal in most cases to concentric peak force in the present study. In contrast, this study found that only SLJ jump height asymmetry was associated with a significantly elevated injury risk in boys, while Fort-Vanmeerhaeghe et al^11^ reported that in both young male and young female athletes, SLJ jump height asymmetries were significantly higher in those who became injured. Interestingly, however in the context of our findings, the difference in the mean percentage of asymmetry between the injured and uninjured groups was larger in male participants (uninjured: 9.7%; injured: 17.1%) than in female participants (uninjured: 7.7%; injured: 12.8%).

There are well-established sex differences in drop jump–landing biomechanics and associations with the risk of anterior cruciate ligament injuries^17,34^ in female athletes. These injuries are, however, uncommon in female ballet dancers^10,31^ (zero incidence in the present study), and therefore, the relevance of this to a preprofessional ballet cohort is questionable. Nonetheless, it is interesting that asymmetry in eccentric phase (downward) variables related to the rapid deceleration of body mass and early landing impact was more robustly associated with the risk of (principally overuse) injuries in female dancers. For example, despite the large loss of participants in the analysis (from n = 107 to n = 56), the RR for eccentric peak force for the dominant left limb rose from 1.45 to 1.72 in female dancers. Whether sex differences in jump biomechanics or neuromuscular and musculotendinous qualities related to deceleration and force attenuation can explain some of the findings in the present study should be examined in further research.

In understanding and interpreting these findings, the substantially different nature of activities carried out by male and female dancers within the balletic training and performance context should also be considered. For instance, female dancers traditionally perform much more work *en pointe* (in a fully plantarflexed position), whereas male dancers traditionally perform more intensive jumping activities.^1,31^ This difference affects injury mechanisms in male and female dancers. Female dancers are more likely to experience overuse foot and ankle injuries, while male dancers suffer more severe traumatic injuries related to jumping and landing.^1,31^

Inherent to the subanalysis separating boys and girls, there was a substantial loss of statistical power, and the sample size may not have been adequate for such an analysis, resulting in a type II error: in particular, when separating left limb– and right limb–dominant students to determine associations with the direction of asymmetry. For instance, in female dancers, significant associations between eccentric RFD asymmetry on the dominant left side were observed, with 10 of the 14 dancers (71%) with high asymmetry becoming injured. In male dancers, despite 9 of the 12 dancers (75%) with high asymmetry in the same variable becoming injured, RRs for the association were not significant because of the lower overall numbers and thus reduced sensitivity (Appendix 3, available online). As such, noted sex differences should not be overinterpreted, and further research with larger sample sizes is needed to confirm the apparent differences.

### Implications for Dance Clinicians

In addition to kinetic asymmetries during the DL-CMJ, higher left limb–dominant jump height asymmetry during the SLJ was associated with the injury risk, although this was not significant in female participants. From a practical perspective, SLJ jump height can be measured using a variety of lower cost devices^21,44^ and therefore can be obtained by practitioners working within less well-funded dance or other sporting institutions without access to force platforms. Given the present and previous evidence,^11,33,36^ in these environments, an assessment of jump height asymmetry during an SLJ might be considered a prudent screening tool, at least in young athletes. Future studies should examine kinetic asymmetries during the SLJ, as well as jump height asymmetries, to determine if asymmetries in aspects of neuromuscular performance are more strongly associated with the injury risk than jump height.

Overall, this study's analysis indicates that kinetic asymmetries during the DL-CMJ may be more strongly associated with the injury risk than SLJ jump height asymmetry specifically, the early concentric (upward) phase variables (concentric impulse 100 ms and concentric impulse part 1), which were the only variables to be significant for both sexes. Furthermore, this detailed analysis identified asymmetries in specific neuromuscular characteristics and phases in the jump-landing movement cycle, insights that may inform more targeted corrective programming. The finding that elevated asymmetry in specific variables in a phase during the DL-CMJ was associated with the injury risk (ie, eccentric RFD and eccentric peak force), while other variables in the same phase (eg, eccentric deceleration impulse and force at 0 velocity) were not, also supports the importance of a comprehensive analysis of DL-CMJ kinetic data to allow the identification of the variables and characteristics that are most strongly associated with the outcome of interest (ie, injury risk).^4,8,40^

### Critique of Analysis Techniques

This study used a moderate threshold to define an injury, an approach chosen so that only injuries affecting participation in dance practices or required more substantial or lengthy rehabilitation were included. Because of the wide range of intensities and skills required during ballet practices, participation in light rehearsals is possible, even when a dancer is suffering a significant injury. Conversely, performing higher intensity activities such as large pirouettes and jumps can be impossible, even with a relatively minor injury. For this reason, injury definitions commonly applied in studies on the injury risk in athletic populations may be inappropriate for the present population.^22^ Therefore, while this threshold does not align with other epidemiological studies on ballet dancers,^13,28^ it was considered the most relevant from a practical perspective within the current population. The inclusion of all lower intensity injuries that may have limited full participation in dance practices would have substantially increased the number of dancers defined as injured (to n = 143) and made the analysis less meaningful.

An asymmetry threshold of ≥Mean Asymmetry % + (0.5*SD) was used to classify elevated asymmetry; while being an arbitrary cut point, it is a statistically derived threshold based on the characteristics of the sample and specific to each variable rather than the predefined asymmetry thresholds of 10% or 15% often employed in asymmetry-risk studies.^18,24,32^≥Mean Asymmetry % + (0.75*SD) and ≥Mean Asymmetry % + (1*SD) cut points (data not shown) were also assessed, with both showing inferior performance considering the RRs and 95% CIs, suggesting that, at least in the present population, the initial cut point was appropriate. The results suggest that cut points for high asymmetry determined using simple descriptive statistics applied to the cohort data were also associated with a meaningful clinical outcome and therefore useful in identifying the injury risk. This is particularly pertinent to preprofessional ballet, for which there are little normative data or prospective research available, and while this approach has also been demonstrated in military cadets,^30^ further research in other athletic groups is warranted to establish if this method can be more widely applied across populations. This approach may, however, be limited to scenarios in which the practitioner has access to a large enough pool of athletes to calculate a representative mean and standard deviation.

### Limitations

This study provides some rationale for the use of jump-based asymmetry screening assessments at the start of preprofessional ballet dancers’ annual training cycle. However, because of the exploratory nature of this investigation, this should be considered the first step in examining potential links between jumping asymmetry and injuries in preprofessional ballet dancers. Because of the considerable number of comparisons made in this trial, there is an increased chance of a type I error in these findings. However, this study does provide detailed evidence for future research within this population, which was previously lacking.

If these prospective findings can be replicated, there is also a lack of clarity on how these factors respond longitudinally and interact with injuries. Various dynamic factors such as maturation levels, energy intake, specific loading, and fatigue may have influenced neuromuscular performance and asymmetries before the injury occurred because the mean time between testing and an injury was 125.6 days (see Table 2). Further analysis investigating how asymmetries respond longitudinally and during dynamic dance activities is warranted. In addition, the generalizability of these findings to other groups is unclear because of the highly specialized training and characteristics of the present population. The association between jump-landing kinetic asymmetries and the injury risk in other groups of youth athletes or dancers should be investigated in future studies using an internal, variable-specific, statistical cut point approach.

## Conclusion

Kinetic asymmetries in the DL-CMJ and SLJ jump height asymmetries were associated with an elevated risk of injuries in preprofessional ballet dancers. Most of these associations were observed in left limb–dominant asymmetries rather than right limb–dominant or absolute asymmetries. This indicates the importance of investigating not only absolute but also left limb– and right limb–dominant asymmetries, as associations would have been missed if directional asymmetries were not evaluated. Sex differences were also observed with these associations. Broadly, for female dancers, asymmetries in the eccentric, and landing phases of the DL-CMJ were more strongly associated with the injury risk, while the association with SLJ jump height asymmetry was attenuated. This study builds on previous research describing the dominance of the right side in ballet practices and performances and provides a starting point for further detailed investigations on links between jumping asymmetry and the injury risk within this population. Should these links be further established, this may provide a rationale for the diversification of ballet practices and the provision of unilateral supplementary training, as well periodic screening for jump-land asymmetries. While neuromuscular asymmetries are only a single component in the complex and multifactorial injury risk picture, this study provides useful insights into a potentially modifiable risk factor that can be screened for in various settings and might be addressed with appropriate training modifications.

## Supplemental Material

sj-pdf-1-ajs-10.1177_03635465231218258 – Supplemental material for Jumping Asymmetries and Risk of Injuries in Preprofessional Ballet Click here for additional data file. Supplemental material, sj-pdf-1-ajs-10.1177_03635465231218258 for Jumping Asymmetries and Risk of Injuries in Preprofessional Ballet by Niall D.H. MacSweeney, Joseph W. Shaw, George P. Simkin, Charles R. Pedlar, Phil D.B. Price, Ryan Mahaffey and Daniel D. Cohen in The American Journal of Sports Medicine
