# Supplementary material for: Jumping Asymmetries and Risk of Injuries in Preprofessional Ballet
Source: Am J Sports Med. 2024 Jan 22;52(2):492–502. doi: 10.1177/03635465231218258 (PMC10838486; doi:10.1177/03635465231218258)
Supplement: sj-pdf-1-ajs-10.1177_03635465231218258 – Supplemental material for Jumping Asymmetries and Risk of Injuries in Preprofessional Ballet [file sj-pdf-1-ajs-10.1177_03635465231218258.pdf]

Appendix 1-Kinetic Variables for Asymmetry Analysis

Variable	Description
Downward phase	
Eccentric Minimum Force	Asymmetry between the lowest force during the downward phase
Eccentric Yielding RFD	Asymmetry between the rate of force development during the eccentric yielding phase (eccentric minimum force to maximum negative velocity)
Eccentric Deceleration RFD	Asymmetry between the rate of force development across the eccentric deceleration phase (maximum negative velocity to zero velocity).
Eccentric Deceleration Impulse	Asymmetry between absolute impulse during the eccentric deceleration phase (maximum negative velocity to zero velocity)
Eccentric Peak Force	Asymmetry between the highest force during the downward phase
Force at Zero Velocity	Asymmetry between force at the moment of zero velocity (maximum negative displacement).
Upward phase	
Concentric Impulse 100ms	Asymmetry between absolute impulse during the first 100ms of the upward phase of the jump.
Concentric Impulse P1	Asymmetry between absolute impulse during the first 50% timewise in the upward phase of the jump.
Concentric Impulse P2	Asymmetry between absolute impulse during the second 50% timewise of the upward phase.
Concentric Peak Force	Asymmetry between the highest force during the upward phase of the jump.
Force at Concentric Peak	Asymmetry between force at the moment of concentric
Power	peak power
Concentric Impulse	Asymmetry between absolute impulse during the upward phase of the jump
Landing Phase	
Landing Impulse 40ms	Asymmetry between absolute impulse during the first 40ms of the landing phase
Landing RFD 40ms	Asymmetry between the rate of force development during the first 40ms of the landing phase

Average Landing RFD	Asymmetry between the rate of force development between the beginning of the landing phase and the landing peak force
Landing Peak Force	Asymmetry between the highest force during landing
Landing Impulse	Asymmetry between absolute impulse during the landing phase

Appendix 2-Sample Asymmetry Classification Calculations

**Variable asymmetry thresholds**	**Con Impulse (Absolute: 7.7, L: 6.5 R: 8.4)**		**Ecc RFD (Absolute: 16, L: 15.4 R: 16.4)**	
**Student**	**Asymmetry (direction, %)**	**Variable Asymmetry Classifications**	**Asymmetry (direction, %)**	**Variable Asymmetry Classifications**
1	R 3.5	Normal Abs<br>Normal R	R 6.9	Normal Abs<br>Normal R
2	R 17.4	High Abs<br>High R	R 42.4	High Abs<br>High R
3	L 8.1	High Abs<br>High L	L 10	Normal Abs<br>Normal L
4	L 0.1	Normal Abs<br>Normal L	L 17	High Abs<br>High L
5	R 3.2	Normal Abs<br>Normal R	L 25.1	High Abs<br>High L

Abs= Absolute; L= Left; R= Right; Con=Concentric; Ecc=Eccentric; RFD=Rate of force development

Sample asymmetry calculations using data from the present sample. Asymmetry classifications across variables and limbs are shown for different students.

High Absolute Asymmetry Calculation Example

$$High\ absolute\ asymmetry\ calculation = Mean\ (BSA\ independent\ of\ side) + (0.5\ X\ Standard\ Deviation\ (BSA\ independent\ of\ side))$$

$$e.g. High\ concentric\ impulse\ asymmetry = 5.5 + (0.5\ X\ 5.5) = 7.7$$

High Left Limbed Asymmetry Calculation Example

$$High\ left\ asymmetry\ calculation = Mean\ (Left\ limbed\ BSA\ only\ ) + (0.5\ X\ Standard\ Deviation\ (Left\ limbed\ BSA\ only))$$

$$e.g. High\ left\ limbed\ concentric\ impulse\ asymmetry = 4.8 + (0.5\ X\ 3.4) = 6.5$$

High Right Limbed Asymmetry Calculation Example

$$High\ right\ asymmetry\ calculation = Mean\ (Right\ limbed\ BSA\ only) + (0.5\ X\ Standard\ Deviation\ (Right\ limbed\ BSA\ only))$$

$$e.g. High\ right\ limbed\ concentric\ impulse\ asymmetry = 6 + (0.5\ X\ 4.8) = 8.4$$

BSA= Bilateral Strength Asymmetry Score

The same process was completed for the sub analysis which split dancers by gender. As a result, asymmetry classifications were always relative the to the specific group investigated. i.e. concentric peak force high asymmetry threshold would be different for absolute, right and left values for each gender.

Appendix 3-Contingency Tables for the Full Sample, Male and Female dancers

Table A1- Contingency Table for DL-CMJ kinetic and SLJ height Asymmetries

Contingency Table		Normal Asymmetry		High Asymmetry		Sensitivity	Specificity
Kinetic Variable	Limb	Healthy	Injured	Healthy	Injured		
DOWNWARD ("*ECCENTRIC*") PHASE							
ECC MINIMUM FORCE	Absolute	92	104	22	24	0.19	0.81
	Left	56	55	7	11	0.17	0.89
	Right	36	47	15	15	0.24	0.71
ECC YIELDING RFD	Absolute	80	97	34	31	0.24	0.70
	Left	34	47	17	14	0.23	0.67
	Right	45	52	18	15	0.22	0.71
ECC DECCEL RFD	Absolute	83	93	31	35	0.27	0.73
	Left	36	44	13	20	0.31	0.73
	Right	47	49	18	15	0.23	0.72
ECC RFD	Absolute	92	98	22	30	0.23	0.81
	**Left**	**44**	**37**	**7**	**19**	**0.34**	**0.86**
	Right	48	59	15	13	0.18	0.76
ECC DECCEL IMPULSE	Absolute	84	102	30	26	0.20	0.74
	Left	37	43	14	14	0.25	0.73
	Right	47	58	16	13	0.18	0.75
ECC PEAK FORCE	Absolute	86	96	28	32	0.25	0.75
	**Left**	**42**	**37**	**9**	**19**	**0.34**	**0.82**
	Right	44	57	19	15	0.21	0.70
UPWARD ("*CONCENTRIC*") PHASE							
CON IMPULSE 100 ms	Absolute	92	98	22	30	0.23	0.81
	**Left**	**44**	**37**	**7**	**19**	**0.34**	**0.86**
	Right	48	59	15	13	0.18	0.76
CON IMPULSE PART 1	Absolute	90	93	24	35	0.27	0.79
	**Left**	**41**	**33**	**9**	**25**	**0.43**	**0.82**
	Right	47	54	17	16	0.23	0.73
CON IMPULSE PART 2	Absolute	87	93	27	35	0.27	0.76
	**Left**	**39**	**29**	**6**	**16**	**0.36**	**0.87**
	Right	51	64	18	19	0.23	0.74
CON PEAK FORCE	**Absolute**	**89**	**86**	**25**	**42**	**0.33**	**0.78**
	**Left**	**40**	**37**	**7**	**23**	**0.38**	**0.85**
	Right	51	48	16	20	0.29	0.76
FORCE @ CON PEAK POWER	Absolute	87	94	27	34	0.27	0.76
	Left	40	34	9	17	0.33	0.82
	Right	45	60	20	17	0.22	0.69
CON IMPULSE	Absolute	89	93	25	35	0.27	0.78
	**Left**	**36**	**35**	**6**	**21**	**0.38**	**0.86**
	Right	56	56	16	16	0.22	0.78

LANDING PHASE							
LANDING IMPULSE 40 ms	**Absolute**	**91**	**87**	**23**	**41**	**0.32**	**0.80**
	Left	41	42	13	17	0.29	0.76
	**Right**	**49**	**46**	**11**	**23**	**0.33**	**0.82**
LANDING RFD 40 ms	**Absolute**	**89**	**85**	**25**	**43**	**0.34**	**0.78**
	Left	42	40	14	20	0.33	0.75
	Right	47	46	11	22	0.32	0.81
AVERAGE LANDING RFD	Absolute	89	94	25	34	0.27	0.78
	Left	39	46	10	20	0.30	0.80
	Right	49	45	16	17	0.27	0.75
LANDING PEAK FORCE	Absolute	90	97	24	31	0.24	0.79
	Left	47	49	9	16	0.25	0.84
	Right	43	48	15	15	0.24	0.74
LANDING IMPULSE	Absolute	87	93	27	35	0.27	0.76
	Left	41	44	14	13	0.23	0.75
	Right	45	46	14	25	0.35	0.76
SINGLE LEG COUNTERMOVEMENT JUMP							
JUMP HEIGHT	Absolute	89	90	25	38	0.30	0.78
	**Left**	**43**	**42**	**7**	**19**	**0.31**	**0.86**
	Right	46	48	18	19	0.28	0.72

Table A2- Contingency Table for DL-CMJ kinetic and SLJ height Asymmetries- Male Dancers

**Contingency Table**		Normal Asymmetry		High Asymmetry		Sensitivity	Specificity
Kinetic Variable	Limb	Healthy	Injured	Healthy	Injured		
DOWNWARD ("*ECCENTRIC*") PHASE							
ECC MINIMUM FORCE	Absolute	39	46	10	19	0.20	0.73
	**Left**	**23**	**25**	**2**	**9**	**0.28**	**0.82**
	Right	16	20	8	11	0.19	0.67
ECC PEAK FORCE	Absolute	36	52	13	13	0.20	0.73
	Left	18	21	4	8	0.28	0.82
	Right	18	29	9	7	0.19	0.67
ECC RFD	Absolute	40	51	9	14	0.22	0.82
	Left	20	20	3	9	0.31	0.87
	Right	20	31	6	5	0.14	0.77
UPWARD ("*CONCENTRIC*") PHASE							
CON IMPULSE 100 ms	Absolute	37	49	12	16	0.25	0.76
	**Left**	**19**	**16**	**3**	**11**	**0.41**	**0.86**
	Right	18	30	9	8	0.21	0.67
CON IMPULSE PART 1	Absolute	37	51	12	14	0.22	0.76
	**Left**	**19**	**19**	**3**	**11**	**0.37**	**0.86**
	Right	18	27	9	8	0.23	0.67

CON IMPULSE PART 2	Absolute	40	48	9	17	0.26	0.82
	Left	18	13	3	6	0.32	0.86
	Right	22	35	6	11	0.24	0.79
CON PEAK FORCE	Absolute	37	44	12	21	0.32	0.76
	**Left**	**18**	**18**	**2**	**12**	**0.40**	**0.90**
	Right	21	26	8	9	0.26	0.72
CON IMPULSE	Absolute	38	49	11	16	0.25	0.78
	**Left**	**18**	**14**	**2**	**10**	**0.42**	**0.90**
	Right	22	32	7	9	0.22	0.76
LANDING PHASE							
LANDING IMPULSE 40 ms	Absolute	39	44	10	21	0.32	0.80
	Left	16	20	5	7	0.26	0.76
	Right	23	24	5	14	0.37	0.82
LANDING RFD 40 ms	Absolute	34	41	15	24	0.37	0.69
	Left	14	17	8	9	0.35	0.64
	Right	20	25	7	14	0.36	0.74
SINGLE LEG COUNTERMOVEMENT JUMP							
JUMP HEIGHT	Absolute	38	47	11	18	0.28	0.78
	**Left**	**21**	**18**	**2**	**10**	**0.36**	**0.91**
	Right	17	29	9	8	0.22	0.65

Table A3- Contingency Table for DL-CMJ kinetic and SLJ height Asymmetries- Female Dancers

Contingency Table		Normal Asymmetry		High Asymmetry		Sensitivity	Specificity
Kinetic Variable	Limb	Healthy	Injured	Healthy	Injured		
DOWNWARD ("*ECCENTRIC*") PHASE							
ECC MINIMUM FORCE	Absolute	53	28	12	5	0.20	0.73
	Left	33	30	5	2	0.28	0.82
	Right	20	7	7	4	0.19	0.67
ECC PEAK FORCE	Absolute	50	44	15	19	0.30	0.77
	**Left**	**24**	**16**	**5**	**11**	**0.41**	**0.83**
	Right	26	28	10	8	0.22	0.72
ECC RFD	Absolute	52	47	13	16	0.25	0.80
	**Left**	**24**	**17**	**4**	**10**	**0.37**	**0.86**
	Right	28	28	9	8	0.22	0.76
UPWARD ("*CONCENTRIC*") PHASE							
CON IMPULSE 100 ms	**Absolute**	**53**	**42**	**12**	**21**	**0.33**	**0.82**
	**Left**	**22**	**15**	**6**	**13**	**0.46**	**0.79**
	Right	28	26	9	9	0.26	0.76
CON IMPULSE PART 1	**Absolute**	**53**	**42**	**12**	**21**	**0.33**	**0.82**
	**Left**	**22**	**14**	**6**	**14**	**0.50**	**0.79**
	Right	29	27	8	8	0.23	0.78
CON IMPULSE PART 2	Absolute	47	45	18	18	0.29	0.72
	**Left**	**21**	**16**	**3**	**10**	**0.38**	**0.88**
	Right	29	29	12	8	0.22	0.71
CON PEAK FORCE	Absolute	52	42	13	21	0.33	0.80
	Left	22	19	5	11	0.37	0.81
	Right	30	22	8	11	0.33	0.79
CON IMPULSE	Absolute	51	44	14	19	0.30	0.78
	Left	18	21	4	11	0.34	0.82
	Right	34	24	9	7	0.23	0.79
LANDING PHASE							
LANDING IMPULSE 40 ms	Absolute	52	43	13	20	0.32	0.80
	Left	25	22	8	10	0.31	0.76
	Right	26	22	6	9	0.29	0.81
LANDING RFD 40 ms	Absolute	55	44	10	19	0.30	0.85
	Left	28	23	6	11	0.32	0.82
	Right	27	21	4	8	0.28	0.87
SINGLE LEG COUNTERMOVEMENT JUMP							
JUMP HEIGHT	Absolute	51	43	14	20	0.32	0.78
	Left	22	24	5	9	0.27	0.81
	Right	29	19	9	11	0.37	0.76
